# Different impact of early and late stages irreversible eye diseases on vision-specific quality of life domains

**DOI:** 10.1038/s41598-022-12425-9

**Published:** 2022-05-19

**Authors:** Preeti Gupta, Eva K. Fenwick, Ryan E. K. Man, Alfred T. L. Gan, Charumathi Sabanayagam, Debra Quek, Chaoxu Qian, Chui Ming Gemmy Cheung, Ching-Yu Cheng, Ecosse L. Lamoureux

**Affiliations:** 1 Singapore Eye Research Institute (SERI) and Singapore National Eye Centre, Population Health, 20 College Rd, The Academia, Discovery Tower Level 6, Singapore, 169856 Singapore; 2 Duke-NUS Medical School, Singapore, Singapore; 3 National University of Singapore, Singapore, Singapore

**Keywords:** Eye diseases, Quality of life

## Abstract

To determine the differential impact of the irreversible eye diseases on vision-related quality of life (VRQoL) in a multi-ethnic Asian population. 2652 participants from the Singapore Epidemiology of Eye Disease Study, with any of the following early and late-stage eye conditions including age-related macular degeneration (AMD, n = 158), diabetic retinopathy (DR, n = 105; non vision threatening [non-VTDR]; VTDR), glaucoma (n = 57) and myopic macular degeneration (MMD, n = 106), or none of the above (controls, 2226 [83.9%]) were included. Rasch-scaled scores of the Emotional well-being Mobility and Reading subscales of the Impact of Vision Impairment (IVI) questionnaire, collectively referred to as “VRQoL” were assessed. Multivariable linear regression analyses and pairwise comparisons adjusting for age, gender, ethnicity, socio-economic status, BMI, smoking, alcohol use, presence of systemic diseases and presenting VI were performed to assess and compare the impact of the presence and severity of each eye condition on the three IVI domains. Multivariable adjusted pairwise comparisons of VRQoL between early stages of the four eye diseases showed no significant differences (all P > 0.05). For late stage diseases, individuals with VTDR had significantly larger decrements in Emotional well-being compared to glaucoma (β − 0.81; 95% CI − 1.47 to − 0.16) and MMD (β − 1.17; 95% CI − 2.16 to − 0.18); and Reading decrements compared to glaucoma (β − 0.66; 95% CI − 1.22 to − 0.11). When compared to late glaucoma, individuals with late AMD (β − 0.76; 95% CI − 1.50 to − 0.01) had significantly larger IVI Mobility subscale decrements. VTDR and late AMD, appear to have the greatest impact on VRQoL, compared to late glaucoma and MMD, suggesting a differential impact of late-stage eye disease categorization on VRQoL.

## Introduction

Global estimates in 2018 suggested that 43.3 million people were blind and 553 million lived with visual impairment (VI)^1^, with Asia alone accounting for ~ 60% of these cases^2^. While cataract and uncorrected refractive error are the two most common causes of vision loss in adults^3^, the associated VI can be corrected via cataract surgery and the dispensing of optical aids, respectively^4,5^. Conversely, the four major causes of *irreversible* VI, including age-related macular degeneration (AMD), diabetic retinopathy (DR), glaucoma, and myopic macular degeneration (MMD)^6^, are on the rise with the rapid ageing of the global population. Current global prevalence estimates were 8.7%^7,8^, 34.6%^9^, 3.5%^10,11^, and 2.1%^12^, for AMD, DR, glaucoma and MMD, respectively, with Asia having the world’s highest proportion of AMD (35%, 59 million)^7^ and glaucoma (60%, 39 million) cases^10^.

These four major irreversible eye conditions have all demonstrated considerable detrimental impact on overall VRQoL and associated domains^13–20^, including functioning^13,14,18,21^, emotional well-being^22^, mobility and independence^23^, when compared to controls. Given their distinct clinical presentations, symptoms, and treatment regimens however, their deleterious impact on VRQoL is also likely to be disparate. Therefore, it is essential to understand the differential impact of these diseases on VRQoL across their severity spectrum to allow clinicians and allied health practitioners to provide more tailored rehabilitative plans for their patients. This is important given that healthcare is increasingly moving towards a more holistic, value-based care model^24^.

In this study, we investigated and compared the impact of the presence and severity of four major irreversible eye diseases, i.e., AMD, DR, glaucoma, and MMD on VRQoL in a multi-ethnic Asian population in Singapore using the Emotional well-being, Mobility and Reading scales of the Impact of Vision Impairment (IVI) questionnaire. Based on the known variations in functional and clinical effects of different eye conditions, we hypothesize that the impact of the four major eye diseases on the three VRQoL outcomes is likely to differ across the different disease severity levels (e.g., early vs. late). Such information could improve patient-physician interaction and assist in the shared decision-making process, leading to better-targeted referral to rehabilitation services. Importantly, our results could serve as a foundation for further research to evaluate the impact of the presence of concomitant ocular disease and associated rehabilitation strategies on an individual’s VRQoL.

## Methods

### Study population and design

The Singapore Epidemiology of Eye Diseases (SEED) Study is a longitudinal population-based study in Singapore that comprises adults from three major ethnicities: Chinese, Malay, and Indian. The methodology of the SEED study has been previously described^25–28^. Briefly, participants aged 40–80+ years residing in the Southwestern part of Singapore were recruited and underwent standardized ocular and systemic examinations, with baseline visits conducted between 2004 and 2011 and 6-year follow-up visits from 2011 to 2017. As the baseline visits in the Malay and Indian populations did not include the IVI questionnaire, we included 3353 Chinese from the baseline visit in year 2009–2011 (response rate 72.8%), 1901 Malays from the 6-year follow up visit in 2011–2013 (response rate 72.1%), and 2200 Indians from the 6-year follow up visit in year 2013–2015 (response rate 75.5%).

Of the 7454 participants, we excluded 1756 subjects with any missing data (vision or eye conditions [n = 1240], systemic health [n = 363], socio-demographics [n = 151], and questionnaire [n = 2]) and 3000 individuals with an eye condition not of interest to this study (under corrected refractive error, cataract and non-diabetic retinopathy). Because the small numbers precluded any meaningful comparison of the impact of single vs. multiple eye diseases on VRQoL domains, we further excluded 46 subjects with more than one eye condition of interest, leaving 2652 participants for the current analyses with either no eye disease or a single eye disease of interest (DR, AMD, glaucoma and MMD). Of these, 94 (3.5%) and 503 (19%) subjects had presenting VI in the better and worse eye, respectively. The study protocol was administered at the research clinic of the Singapore Eye Research Institute. All protocols followed the principles of the Declaration of Helsinki and received approval by the SingHealth Institutional Review Board. Written informed consent from participants was obtained prior to participation in the study.

### Clinical examination and assessment of eye diseases

All participants underwent a comprehensive ophthalmic examination, which included visual acuity (VA) testing, colour fundus photography and a detailed clinical slit-lamp examination.

#### Vision assessment

VA was measured using a logarithm of the minimum angle of resolution (LogMAR) vision chart (Lighthouse International) at a distance of 4 m. If no numbers were read at 4 m, the participant was moved to 3, 2, and then 1 m. If no numbers were identified on the chart, presenting VA (PVA) was assessed as counting fingers, hand movements, perception of light, or no perception of light. PVA was measured in the left and right eyes separately with patients wearing their usual habitual optical correction (e.g., spectacles or contact lenses). PVA in the better eye was used in the current study as it best represents the role of VI in participants’ performance of day-to-day tasks^29^, and as we have shown in an earlier study that the VRQoL decrements from presenting better-eye VA loss most closely resembles that resulting from binocular VA deficits^30^. Better-eye VI was defined as a LogMAR score of > 0.3 (< 6/12) in the better-seeing eye.

#### Assessment and definitions of AMD, DR, glaucoma and MMD

Fundus photographs were taken of each participant using a digital retinal camera (Canon CR-DGi with digital 10D SLR camera backing; Canon) following pupil dilation. Two-field color photographs were taken for each eye—one centered on the optic disc and the other on the fovea—according to the Early Treatment for Diabetic Retinopathy Study guidelines. The better eye was used for analysis, defined as the eye with less severe disease level or, by convention, the right eye in patients who had same severity level for both eyes.

*AMD* was graded from retinal photographs for presence and severity by trained graders using the modified Wisconsin Age-Related Maculopathy Grading System^31^. *Early* *AMD* was defined as the presence of any soft drusen and increased or decreased retinal pigment or as the presence of large soft drusen (≥ 125 μm in diameter) with a large drusen area greater than 500 μm in diameter or large (≥ 125 μm) indistinct soft drusen in the absence of signs of late AMD. *Late* *AMD* was defined as the presence of geographic atrophy or exudative macular degeneration or both^32^.

In individuals with diabetes mellitus, *DR* was graded for presence and severity using the modified Airlie House classification system into no (level 10–15), early i.e. non vision-threatening (non-VTDR; including minimal [level 20], mild [level 35] and/or moderate [level 43–47] DR) and late i.e. VTDR (including severe non-proliferative retinopathy [NPDR; level 53], proliferative retinopathy [PDR; level 61–90], and/or clinically significant macular edema [CSME]) using data from the worse eye^33,34^. Any DR was defined as presence of at least minimal DR.

Glaucoma was defined using the International Society of Geographic and Epidemiological Ophthalmology scheme^35^, based on findings from gonioscopy, optic disc characteristics, and visual fields results. Glaucoma clinical severity was based on mean deviation (MD) thresholds from visual fields as early-stage (MD ≥ − 12.00 dB), and late-stage (− 12.01 to − 20.00 dB) glaucoma, respectively.

Myopia was defined as spherical equivalent ≤ − 0.5 diopters. Based on the International META-PM classification^36^, the presence of MMD was defined and classified into the following categories: no macular lesions (category 0); tessellated fundus only (category 1); diffuse chorioretinal atrophy (category 2); patchy chorioretinal atrophy (category 3); and macular atrophy (category 4). “Plus” lesions, which supplemented the Meta-PM categories, comprised lacquer cracks, choroidal neovascularization, and Fuchs spot. Based on fundus photograph grading, an eye was considered to have MMD if Meta-PM category 2, 3, 4, or any “plus” lesion, was observed^37^. Eyes with META-PM categories 0 and/or 1 were considered as non-MMD myopia. Eyes were considered to have early MMD when Meta-PM category 2 was observed, whereas presence of Meta-PM categories 3 or 4 was considered as late MMD.

### Assessment of VRQoL

The 32-item IVI questionnaire was administered to participants by trained multi-lingual interviewers in English, Malay, Tamil, or Mandarin. If a participant spoke more than one language, the interview (including the IVI questionnaire) was administered in their preferred language. The IVI comprises three subscales (domains) including Emotional Well-being (“Emotional”), Mobility and Independence (“Mobility”); and Reading and Accessing Information (“Reading”)^38,39^. Having undergone rigorous psychometric assessment in clinical and population-based samples, the instrument’s validity and reliability have been previously demonstrated^22,23,40,41^. Rasch analysis was undertaken to assess the psychometric properties of the three IVI domains separately in the present sample using the Andrich rating scale model^42^ with Winsteps software (version 4.6.0, Chicago, Illinois, USA; https://www.winsteps.com/index.htm). The summary of Rasch fit statistics for this study is presented in Supplementary Table S1. Following satisfactory fit to the Rasch model, the ordinal raw scores were transformed to estimates of interval measures in units of logits, which were used in subsequent statistical parametric analyses^43^.

### Assessment of other covariates

Trained interviewers fluent in English, Malay, Tamil, and Mandarin administered questionnaires to collect sociodemographic characteristics, family and medical history, and lifestyle factors. Two measurements of systolic blood pressure (SBP) and diastolic blood pressure (DBP) were taken using a digital automatic BP monitor (Dinamap Pro Series DP110X-RW; GE Medical Systems Information Technologies, Inc), and a third measurement was obtained if the 2 previous SBP or DBP readings differed by more than 10 or 5 mm Hg, respectively. The mean of these measurements was used in analyses. Height was measured using a wall-mounted, adjustable measuring scale, and weight was measured with a calibrated scientific weight scale. Body mass index was calculated as weight in kilograms divided by height in meters squared. Individuals were classified as underweight if they had BMI < 18.5 kg/m^2^, normal weight if they had BMI ≥ 18.5 but < 25 kg/m^2^, overweight if they had BMI ≥ 25 but < 30 kg/m^2^ and obese if they had BMI ≥ 30 kg/m^2^^44^. Blood samples were collected for hemoglobin A1c, random glucose, and total and low-density lipoprotein and high-density lipoprotein cholesterol measurements. Low socioeconomic status (SES) was defined as primary or lower education, individual monthly income < SGD$2000, and living in a1-2 room apartment or smaller^27,45^.

### Statistical analyses

All analyses were conducted with STATA version 16 (Statacorp, TX, USA). Patient-specific analysis data were used. Participants’ details were summarized using means (standard deviation [SD]) for continuous variables and N (%) for categorical variables. We compared the three VRQoL domains (Emotional well-being, Mobility and Reading) between categories of socio-demographical, systemic and vision variables using analysis of variance (ANOVA). Multiple linear regression models were used to determine associations between the presence of each eye disease and their respective severity categories (using data from the worse eye) and the three VRQoL domains, adjusting for traditional confounders of VRQoL. Potential confounders included age, gender, ethnicity (Chinese, Indians and Malays), education level (primary or lower and secondary or higher), monthly income (< S$2000 and ≥ S$2000), housing (3–4 room or less and 5 room house or private)^44^, BMI, smoking status, alcohol use, presence of any systemic disease (diabetes mellitus, arterial hypertension, hyperlipidemia, cardiovascular disease [CVD− defined as self-reported history of stroke, myocardial infarction, or angina]^46^, and chronic kidney disease [CKD– defined as an estimated glomerular filtration rate < 60 ml/min/1.73 m^2^ using the Chronic Kidney Disease Epidemiology Collaboration equation)^47^, and presenting VI. Beta coefficients for each disease were reported in reference to the control group without any eye disease and are interpreted as the adjusted difference between individuals solely with the eye disease in question and those without any eye disease. We also reported absolute adjusted marginal means for each group and beta as a percentage of the marginal mean in controls—this is interpretable as the percentage reduction in mean VRQoL comparing individuals with a particular eye disease against those without eye disease. Importantly, in order to determine if there were any differences in VRQoL decrements between the four eye diseases of interest, we performed pairwise comparisons of VRQoL decrements between eye diseases, grouped by early and late-stage disease. For example, we compared the beta coefficient for early glaucoma with non-VTDR, and the beta coefficient for VTDR with late AMD. Beta coefficients were reported with 95% confidence intervals along with P values that are considered statistically significant if < 0.05.

## Results

### Sociodemographic and clinical characteristics

The mean (SD) age of the 2652 participants was 57.0 (8.8) years, 1283 (48.4%) were male, and 1615 (60.9%) were Chinese (Table 1). A total of 1954 (73.7%) individuals had at least one of the 5 systemic diseases, which included diabetes mellitus, arterial hypertension, hyperlipidemia, CVD or CKD. Participants either had a single eye condition: AMD (any n = 158 [6.0%]; early, n = 115 [5.7%]; late, 7 [0.3%]), DR (any n = 105 [4.0%]; non-VTDR, 81 [3.1%]; VTDR 24 [0.9%]), glaucoma (any n = 57 [2.1%]; early, 31 [1.2%]; late 26 [1.0%]), MMD (any n = 106 [4.0%]; early, 99 [3.7%]; late, 7 [0.3%]), or no eye condition (controls, n = 2226 [83.9%]). Ninety-four (3.5%) and 503 (19%) participants had presenting VI in the better and the worse eye, respectively. The mean ± SD of presenting VA in the better and worse eye was 0.09 ± 0.11 and 0.21 ± 0.22, respectively. The mean ± SD of the mean deviation among 57 subjects with glaucoma with reliable visual fields results was − 7.22 ± 6.29. Participants’ overall mean score on the Emotional well-being, Mobility and Reading domains was 5.45 ± 1.20 (range − 6.15 to 6.00), 5.47 ± 0.92 (range − 1.64 to 5.75) and 5.28 ± 1.02 (range − 0.78 to 5.67) logits, respectively.

**Table 1:** Comparison of IVI scores by sociodemographic and clinical characteristics (N = 2652).

Baseline characteristic	n (%)	Emotional		Mobility		Reading	
		Mean ± SD	% reduction	Mean ± SD	% reduction	Mean ± SD	% reduction
**Age, year**							
40–49	640 (24.1)	5.34 ± 1.18	0.0	5.55 ± 0.70	0.0	5.23 ± 0.98	0.0
50–59	1209 (45.6)	5.49 ± 1.22	2.8	5.55 ± 0.75	0.0	5.41 ± 0.84	3.4
60–69	536 (20.2)	5.54 ± 1.07	3.9	5.42 ± 0.97	− 2.4	5.28 ± 1.02	1.0
70+	267 (10.1)	5.38 ± 1.33	0.8	4.94 ± 1.56	− 11.0	4.77 ± 1.54	− 8.8
		**P = 0.011**		**P < 0.001**		**P < 0.001**	
**Gender**							
Male	1283 (48.4)	5.49 ± 1.18	0.0	5.50 ± 0.85	0.0	5.29 ± 0.99	0.0
Female	1369 (51.6)	5.42 ± 1.21	− 1.3	5.43 ± 0.97	− 1.2	5.26 ± 1.04	− 0.6
		P = 0.135		P = 0.063		P = 0.419	
**Race**							
Malay	491 (18.5)	5.69 ± 0.99	0.0	5.40 ± 1.09	0.0	5.25 ± 1.09	0.0
Indian	546 (20.6)	5.71 ± 1.15	0.3	5.58 ± 0.77	3.4	5.48 ± 0.81	4.2
Chinese	1615 (60.9)	5.29 ± 1.24	− 7.0	5.45 ± 0.90	0.9	5.21 ± 1.04	− 0.8
		**P < 0.001**		**P = 0.002**		**P < 0.001**	
**Educational level**							
Secondary or higher	1446 (54.5)	5.44 ± 1.19	REF	5.54 ± 0.77	REF	5.33 ± 0.93	REF
Primary or below	1206 (45.5)	5.46 ± 1.20	0.4	5.38 ± 1.06	− 3.0	5.21 ± 1.10	− 2.4
		P = 0.620		**P < 0.001**		**P = 0.001**	
**Monthly income**							
≥ S$2000	1024 (38.6)	5.49 ± 1.09	REF	5.59 ± 0.63	REF	5.38 ± 0.85	REF
< S$2000	1628 (61.4)	5.43 ± 1.26	− 1.2	5.38 ± 1.05	− 3.7	5.21 ± 1.10	− 3.1
		P = 0.184		**P < 0.001**		**P < 0.001**	
**Housing**							
5 room house or private	1086 (41.0)	5.45 ± 1.11	REF	5.51 ± 0.82	REF	5.30 ± 0.99	REF
3–4 room or less	1566 (59.0)	5.45 ± 1.25	− 0.1	5.43 ± 0.98	− 1.4	5.26 ± 1.03	− 0.8
		P = 0.901		**P = 0.034**		P = 0.281	
**Lifestyle factors**							
BMI category							
Underweight	98 (3.7)	5.37 ± 1.20	0.0	5.57 ± 0.74	0.0	5.21 ± 1.05	0.0
Normal	1370 (51.7)	5.37 ± 1.28	0.1	5.44 ± 0.95	− 2.2	5.24 ± 1.05	0.4
Overweight	886 (33.4)	5.52 ± 1.12	2.9	5.47 ± 0.92	− 1.9	5.31 ± 0.99	1.9
Obese	298 (11.2)	5.64 ± 0.94	5.2	5.53 ± 0.79	− 0.8	5.37 ± 0.90	3.0
		**P = 0.001**		P = 0.346		P = 0.099	
Smoking status							
Never	1962 (74.0)	5.45 ± 1.17	0.0	5.48 ± 0.90	0.0	5.30 ± 0.99	0.0
Current	371 (14.0)	5.43 ± 1.30	− 0.4	5.52 ± 0.72	0.8	5.24 ± 1.02	− 1.2
Past	319 (12.0)	5.47 ± 1.22	0.4	5.33 ± 1.14	− 2.7	5.17 ± 1.16	− 2.5
		P = 0.888		**P = 0.013**		P = 0.075	
Alcohol use							
No	2425 (91.4)	5.47 ± 1.18	REF	5.46 ± 0.93	REF	5.27 ± 1.02	REF
Yes	227 (8.6)	5.28 ± 1.31	− 3.4	5.53 ± 0.72	1.4	5.34 ± 0.91	1.4
		**P = 0.024**		P = 0.248		P = 0.291	
**Comorbid conditions**							
Diabetes							
No	2080 (78.4)	5.44 ± 1.18	REF	5.48 ± 0.87	REF	5.27 ± 1.01	REF
Yes	572 (21.6)	5.49 ± 1.26	0.8	5.41 ± 1.05	− 1.3	5.28 ± 1.05	0.2
		P = 0.425		P = 0.113		P = 0.828	
Arterial hypertension							
No	1179 (44.5)	5.40 ± 1.22	REF	5.53 ± 0.76	REF	5.31 ± 0.95	REF
Yes	1473 (55.5)	5.49 ± 1.17	1.8	5.41 ± 1.02	− 2.1	5.25 ± 1.06	− 1.1
		**P = 0.040**		**P = 0.001**		P = 0.152	
Hyperlipidaemia							
No	1416 (53.4)	5.46 ± 1.18	REF	5.51 ± 0.78	REF	5.30 ± 0.95	REF
Yes	1236 (46.6)	5.44 ± 1.21	− 0.3	5.41 ± 1.05	− 1.9	5.25 ± 1.08	− 0.9
		P = 0.758		**P = 0.004**		P = 0.222	
Cardiovascular disease							
No	2483 (93.6)	5.46 ± 1.18	REF	5.49 ± 0.87	REF	5.30 ± 0.97	REF
Yes	169 (6.4)	5.35 ± 1.39	− 2.0	5.16 ± 1.37	− 6.0	4.91 ± 1.44	− 7.4
		P = 0.260		**P < 0.001**		**P < 0.001**	
Chronic kidney disease							
No	2504 (94.4)	5.46 ± 1.18	REF	5.51 ± 0.82	REF	5.31 ± 0.96	REF
Yes	148 (5.6)	5.35 ± 1.38	− 2.0	4.75 ± 1.75	− 13.7	4.73 ± 1.61	− 10.9
		P = 0.279		**P < 0.001**		**P < 0.001**	
Any systemic disease							
No	698 (26.3)	5.38 ± 1.27	REF	5.54 ± 0.72	REF	5.31 ± 0.94	REF
Yes	1954 (73.7)	5.48 ± 1.17	1.7	5.44 ± 0.97	− 1.9	5.26 ± 1.04	− 0.8
		P = 0.079		**P = 0.009**		P = 0.342	
**Eye conditions**							
None	2226 (83.9)	5.49 ± 1.11	REF	5.50 ± 0.83	REF	5.30 ± 0.96	REF
Non-VTDR	81 (3.1)	5.55 ± 1.06	1.1	5.26 ± 1.37	− 4.5	5.24 ± 1.23	− 1.2
VTDR	24 (0.9)	4.40 ± 2.81	− 20.0	4.84 ± 1.77	− 12.1	4.58 ± 1.85	− 13.6
Early AMD	151 (5.7)	5.35 ± 1.55	− 2.6	5.33 ± 1.15	− 3.1	5.18 ± 1.18	− 2.3
Late AMD	7 (0.3)	4.92 ± 1.88	− 10.5	4.55 ± 1.58	− 17.3	5.07 ± 1.37	− 4.3
Early glaucoma	31 (1.2)	5.12 ± 1.61	− 6.9	5.25 ± 1.38	− 4.5	5.25 ± 1.31	− 1.0
Late glaucoma	26 (1.0)	5.25 ± 1.49	− 4.5	5.20 ± 1.43	− 5.6	5.16 ± 1.33	− 2.7
Early MMD	99 (3.7)	5.02 ± 1.34	− 8.6	5.39 ± 1.04	− 2.1	5.08 ± 1.19	− 4.3
Late MMD	7 (0.3)	5.37 ± 1.08	− 2.3	4.93 ± 1.41	− 10.5	4.78 ± 0.80	− 9.8
		**P < 0.001**		**P < 0.001**		**P = 0.009**	
**Presenting VI (better eye)**							
No	2558 (96.5)	5.47 ± 1.16	REF	5.48 ± 0.87	REF	5.30 ± 0.97	REF
Yes	94 (3.5)	5.05 ± 1.85	− 7.6	4.94 ± 1.67	− 10.0	4.60 ± 1.68	− 13.3
		**P = 0.001**		**P < 0.001**		**P < 0.001**	
**Presenting VI (worse eye)**							
No	2149 (81.0)	5.52 ± 1.10	REF	5.53 ± 0.79	REF	5.36 ± 0.90	REF
Yes	503 (19.0)	5.18 ± 1.52	− 6.1	5.19 ± 1.29	− 6.1	4.94 ± 1.35	− 7.8
		**P < 0.001**		**P < 0.001**		**P < 0.001**	

Systemic diseases include: diabetes mellitus, arterial hypertension, hyperlipidaemia, cardio vascular disease and chronic kidney disease.

Significant values are in [bold].

*BMI* body mass index, *VTDR* vision-threatening diabetic retinopathy, *AMD* age related macular degeneration, *MMD* myopic macular degeneration, *VI* visual impairment, *REF* reference.

Presence of most ocular conditions and presenting VI (better and worse eye) were associated with significantly lower vision-specific Emotional well-being, Mobility and Reading scores (Table 1).

### Association between the presence of any eye diseases and VRQoL

After multivariable adjustment, compared to those without any eye disease, Emotional well-being was significantly reduced in all four eye diseases, with reductions ranging from 4.7% (β − 0.26; 95% CI − 0.45 to − 0.06) for any AMD to 7.1% (β − 0.39; 95% CI − 0.70 to − 0.08) for any glaucoma. However, Mobility and Reading were significantly lower only in individuals with any DR (Mobility: 5.6%; β − 0.30; 95% CI − 0.48 to − 0.13; Reading: 4.3%; β − 0.23; 95% CI − 0.43 to − 0.03; Table 2, Model 1). Results were unchanged after adjusting for presenting VI (Table 2, Model 2), although the associations between any four eye diseases and Emotional well-being, and any DR and Mobility were slightly attenuated but still significant; while the association between any DR and Reading became insignificant. Pairwise comparisons were not performed here as VRQoL differences between diseases would be confounded by the relative distribution of early and late stage of these diseases.

**Table 2:** Multivariable-adjusted association between eye disease and IVI scores in a multiple linear regression model.

	Emotional				Mobility				Reading			
	Mean ± SE	Beta (95% CI)	P	%	Mean ± SE	Beta (95% CI)	P	%	Mean ± SE	Beta (95% CI)	P	%
**Model 1#**												
None	5.50 ± 0.03	Reference			5.48 ± 0.02	Reference			5.29 ± 0.02	Reference		
DR	5.16 ± 0.12	− 0.34 (− 0.58 to − 0.11)	**0.004**	− 6.19	5.18 ± 0.09	− 0.30 (− 0.48 to − 0.13)	**0.001**	− 5.56	5.06 ± 0.10	− 0.23 (− 0.43 to − 0.03)	**0.024**	− 4.35
AMD	5.25 ± 0.10	− 0.26 (− 0.45 to − 0.06)	**0.010**	− 4.67	5.40 ± 0.07	− 0.08 (− 0.23 to 0.07)	0.290	− 1.46	5.24 ± 0.08	− 0.05 (− 0.21 to 0.12)	0.585	− 0.87
Glaucoma	5.11 ± 0.16	− 0.39 (− 0.70 to − 0.08)	**0.014**	− 7.12	5.34 ± 0.12	− 0.15 (− 0.39 to 0.09)	0.220	− 2.71	5.29 ± 0.13	0.00 (− 0.26 to 0.27)	0.985	0.05
MMD	5.14 ± 0.12	− 0.37 (− 0.60 to − 0.13)	**0.002**	− 6.66	5.50 ± 0.09	0.02 (− 0.16 to 0.20)	0.843	0.33	5.20 ± 0.10	− 0.09 (− 0.29 to 0.11)	0.380	− 1.70
**Model 1 + PVI**												
None	5.50 ± 0.03	Reference			5.48 ± 0.02	Reference			5.29 ± 0.02	Reference		
DR	5.18 ± 0.12	− 0.32 (− 0.55 to − 0.08)	**0.008**	− 5.79	5.20 ± 0.09	− 0.29 (− 0.46 to − 0.11)	**0.002**	− 5.22	5.09 ± 0.10	− 0.20 (− 0.40 to 0.00)	0.051	− 3.75
AMD	5.25 ± 0.10	− 0.25 (− 0.45 to − 0.06)	**0.011**	− 4.57	5.41 ± 0.07	− 0.07 (− 0.22 to 0.07)	0.321	− 1.37	5.25 ± 0.08	− 0.04 (− 0.20 to 0.13)	0.655	− 0.71
Glaucoma	5.10 ± 0.16	− 0.40 (− 0.71 to − 0.09)	**0.012**	− 7.25	5.33 ± 0.12	− 0.15 (− 0.39 to 0.08)	0.202	− 2.82	5.28 ± 0.13	− 0.01 (− 0.27 to 0.26)	0.956	− 0.14
MMD	5.17 ± 0.12	− 0.33 (− 0.57 to − 0.10)	**0.005**	− 6.09	5.53 ± 0.09	0.04 (− 0.14 to 0.22)	0.626	0.81	5.24 ± 0.10	− 0.04 (− 0.25 to 0.16)	0.663	− 0.84

Systemic diseases include diabetes mellitus, arterial hypertension, hyperlipidaemia, CVD and CKD. *BMI* body mass index, *CVD* cardiovascular disease, *CKD* chronic kidney disease, *PVI* presenting visual impairment, *DR* diabetic retinopathy, *AMD* age related macular degeneration, *MMD* myopic macular degeneration.

Significant values are in [bold].

^#^Adjusted for age, gender, race, education, income, housing, BMI, smoking status, alcohol use and presence of any systemic disease.

### Association between eye disease severity and VRQoL

#### Early stage

For early-stage eye disease compared to those with no eye disease, we found significantly lower Emotional well-being for people with early AMD (4.2%; β − 0.23; 95% CI − 0.43 to − 0.03), early glaucoma (8.4%; β − 0.46; 95% CI − 0.88 to − 0.04) and early MMD (6.6%; β − 0.36; 95% CI − 0.61 to − 0.12). Mobility and Reading were not associated with any early-stage eye disease. Multivariable adjusted pairwise comparisons of VRQoL outcomes between early stages of the four eye diseases showed no significant differences (all P > 0.05; Table 3).

**Table 3:** Multivariable-adjusted pairwise comparisons of each IVI domain between early stage eye diseases.

Reference group	Beta coefficient# (95% CI), P value			
	Early MMD	Non-VTDR	Early AMD	Early Glaucoma
**Emotional**				
None	**− 0.36 (− 0.61 to − 0.12)** **P = 0.003**	− 0.08 (− 0.34 to 0.18) P = 0.551	**− 0.23 (− 0.43 to − 0.03)** **P = 0.024**	**− 0.46 (− 0.88 to − 0.04)** **P = 0.031**
Early MMD		0.28 (− 0.07 to 0.63) P = 0.114	0.14 (− 0.17 to 0.44) P = 0.379	− 0.10 (− 0.57 to 0.38) P = 0.693
Non-VTDR			− 0.15 (− 0.47 to 0.17) P = 0.361	− 0.38 (− 0.87 to 0.11) P = 0.127
Early AMD				− 0.23 (− 0.69 to 0.22) P = 0.319
**Mobility**				
None	0.07 (− 0.12 to 0.25) P = 0.464	− 0.19 (− 0.39 to 0.01) P = 0.059	− 0.04 (− 0.19 to 0.11) P = 0.627	− 0.18 (− 0.50 to 0.14) P = 0.261
Early MMD		− 0.26 (− 0.53 to 0.00) P = 0.054	− 0.11 (− 0.34 to 0.12) P = 0.363	− 0.25 (− 0.61 to 0.11) P = 0.174
Non-VTDR			0.16 (− 0.09 to 0.40) P = 0.207	0.01 (− 0.36 to 0.38) P = 0.953
Early AMD				− 0.14 (− 0.49 to 0.20) P = 0.412
**Reading**				
None	− 0.04 (− 0.24 to 0.17) P = 0.737	− 0.06 (− 0.29 to 0.16) P = 0.579	− 0.03 (− 0.20 to 0.14) P = 0.731	− 0.01 (− 0.37 to 0.34) P = 0.935
Early MMD		− 0.03 (− 0.33 to 0.27) P = 0.853	0.01 (− 0.25 to 0.26) P = 0.965	0.02 (− 0.38 to 0.43) P = 0.920
Non-VTDR			0.03 (− 0.24 to 0.30) P = 0.805	0.05 (− 0.37 to 0.46) P = 0.817
Early AMD				0.01 (− 0.37 to 0.40) P = 0.940

Adjusted for age, gender, race, education, income, housing, BMI, smoking, alcohol, any systemic disease & PVI better eye.

Systemic diseases include diabetes mellitus, arterial hypertension, hyperlipidaemia, CVD & CKD.

Significant values are in [bold].

*BMI* body mass index, *CVD* cardiovascular disease, *CKD* chronic kidney disease, *PVI* presenting visual impairment, *VTDR* vision threatening diabetic retinopathy, *AMD* age related macular degeneration, *MMD* myopic macular degeneration.

#### Late stage

For late-stage disease compared to controls, VTDR had the highest decrement in Emotional well-being (20.6% [β − 1.13; 95% CI − 1.61 to − 0.66]) and Reading scores (12.5% [β − 0.66; 95% CI − 1.07 to − 0.26]). Late AMD was associated with the highest decrement in Mobility compared to controls, followed by VTDR (15.9% [β − 0.87; 95% CI − 1.54 to − 0.21] vs 11.1% [β − 0.61; 95% CI − 0.97 to − 0.25]). In contrast, late-stage glaucoma and MMD were not associated with any VRQoL decrements as captured by the IVI scale. Pairwise comparisons of VRQoL outcomes between late stages of the four eye diseases showed that individuals with VTDR had significantly larger decrements in Emotional well-being compared to those with late-stage glaucoma (β − 0.81; 95% CI − 1.47 to − 0.16) and late MMD (β − 1.17; 95% CI − 2.16 to − 0.18), and greater Reading decrements compared to late-stage glaucoma (β − 0.66; 95% CI − 1.22 to − 0.11). Individuals with late AMD had significantly larger IVI Mobility decrements than late glaucoma (β − 0.76; 95% CI − 1.50 to − 0.01; Table 4).

**Table 4:** Multivariable-adjusted pairwise comparisons of each IVI domain between late stage eye diseases.

Reference group	Beta coefficient# (95% CI), P value			
	Late MMD	Late Glaucoma	Late AMD	VTDR
**Emotional**				
None	0.03 (− 0.84 to 0.91) P = 0.939	− 0.32 (− 0.78 to 0.14) P = 0.169	− 0.69 (− 1.56 to 0.18) P = 0.122	**− 1.13 (− 1.61 to − 0.66)** **P < 0.001**
Late MMD		− 0.35 (− 1.34 to 0.63) P = 0.478	− 0.72 (− 1.95 to 0.51) P = 0.250	**− 1.17 (− 2.16 to − 0.18)** **P = 0.021**
Late glaucoma			− 0.37 (− 1.35 to 0.61) P = 0.462	**− 0.81 (− 1.47 to − 0.16)** **P = 0.015**
Late AMD				− 0.45 (− 1.43 to 0.54) P = 0.375
**Mobility**				
None	− 0.32 (− 0.99 to 0.34) P = 0.343	− 0.12 (− 0.46 to 0.23) P = 0.512	**− 0.87 (− 1.54 to − 0.21)** **P = 0.010**	**− 0.61 (− 0.97 to − 0.25)** **P = 0.001**
Late MMD		0.21 (− 0.54 to 0.95) P = 0.589	− 0.55 (− 1.49 to 0.39) P = 0.251	− 0.29 (− 1.04 to 0.47) P = 0.453
Late glaucoma			**− 0.76 (− 1.50 to − 0.01)** **P = 0.048**	− 0.49 (− 1.00 to 0.01) P = 0.052
Late AMD				0.26 (− 0.49 to 1.01) P = 0.498
**Reading**				
None	− 0.21 (− 0.95 to 0.53) P = 0.577	0.00 (− 0.38 to 0.39) P = 0.984	− 0.20 (− 0.94 to 0.55) P = 0.604	**− 0.66 (− 1.07 to − 0.26)** **P = 0.001**
Late MMD		0.22 (− 0.62 to 1.05) P = 0.613	0.02 (− 1.03 to 1.06) P = 0.977	− 0.45 (− 1.29 to 0.39) P = 0.296
Late glaucoma			− 0.20 (− 1.03 to 0.63) P = 0.639	**− 0.66 (− 1.22 to − 0.11)** **P = 0.020**
Late AMD				− 0.46 (− 1.30 to 0.38) P = 0.278

Adjusted for age, gender, race, education, income, housing, BMI, smoking, alcohol, any systemic disease & PVI better eye.

Systemic diseases include diabetes mellitus, arterial hypertension, hyperlipidaemia, CVD & CKD.

Significant values are in [bold].

*BMI* body mass index, *CVD* cardiovascular disease, *CKD* chronic kidney disease, *PVI* presenting visual impairment, *VTDR* vision threatening diabetic retinopathy, *AMD* age related macular degeneration, *MMD* myopic macular degeneration.

Supplementary Tables S2–S4 shows the power to detect effect estimates for each eye condition with the current sample size, as well as the sample size needed for each eye condition using study effect estimates with 80% power and 5% significance level. We have also included unadjusted association between eye disease severity and VRQoL as well as multivariable-adjusted (adjusting for fewer variables including age, gender, ethnicity, low SES, BMI, current smoking and presence of systemic diseases) association between eye disease severity and VRQoL in Supplementary Tables S5 to S8.

## Discussion

In our large, population-based study in multi-ethnic Asian adults living in Singapore, we found a distinctive impact of the early and late stages irreversible eye diseases on vision-specific Emotional well-being, Mobility and Reading. Overall, the presence of any of the four eye diseases, were independently and significantly associated with the decrements in Emotional well-being, with DR additionally associated with a decrement in the Mobility domain, compared to controls. In terms of disease severity comparison, there was no difference in any of the VRQoL outcomes between early-stage eye diseases; while DR followed by AMD, appeared to be the conditions with the greatest impact on VRQoL in late-stage disease. In contrast, late-stage glaucoma and MMD were not associated with any VRQoL decrements as captured by the IVI scale. Our results suggest a variable impact of late-stage eye diseases on VRQoL, reinforcing the need for more holistic rehabilitative interventions, including the use of mobility and low vision aids and counselling, in the management of individuals suffering from these advanced ocular disorders. Importantly, these differential associations also suggest the need for better patient-reported outcome measures (PROMs) for diseases with similar visual function impairments to more precisely quantify the impact of specific eye diseases on QoL across the disease spectrum, possibly leading to better tailored intervention strategies.

Our finding of no difference in any of the VRQoL outcomes between early-stage eye diseases is not unexpected, as early lesions in these diseases such as small size drusen, retinal pigmented epithelium depigmentation, lacquer cracks, and mild visual field loss do not substantially affect VA and other measures of the visual function system such as contrast sensitivity, colour discrimination, thereby maintaining the patient’s ability to carry out these visual tasks. Our findings reinforces current medical treatment guidelines recommending observation with/without standard medical pharmacotherapy for individuals with early AMD, DR, MMD and glaucoma.

For the late stages of the four diseases included in these analyses, VTDR and AMD appear to have the greatest impact on VRQoL, compared to other late-stage eye diseases. The Reading decrements in persons with VTDR may result from vascular changes such as macular edema, neovascularization and contraction of accompanying fibrous tissue that may distort the retina leading to vitreous hemorrhage and retinal detachment causing irreversible central vision loss^48^. In addition, reduced Emotional well-being in those with VTDR could likely be due to the need for repeated anti-VEGF injections, initially at monthly intervals and the associated cost. We are confident that the *ophthalmic* impact of VTDR on emotional well-being, and not the other potential sources of poor mental health, will have accurately been captured by the IVI because of the way the questions are phrased. There is an initial preceding statement: think about how YOUR eyesight has made you FEEL in the PAST MONTH; and each item has embedded in it “because of your eyesight.” Both of these factors focus the respondent to only consider ophthalmic issues affecting emotional well-being rather than other factors. The IVI Emotional scale does not purport to provide a clinical diagnosis of depression/anxiety but has been repeatedly demonstrated to be a valid and reliable subscale for the distinct construct of emotional well-being (sometimes also referred to as vision-specific distress)^49^. Importantly, those with late-stage AMD reported the largest reduction in mobility, possibly because of geographic atrophy as part of late-stage AMD which leads to scotoma including peripheral, resulting in loss of mobility and independence^50^. Our study re-emphasizes the need for preventative strategies to slow the progression to VTDR and late-stage AMD where the VRQoL deficit is considerable and for interventions with a strong focus on improving mobility, reading and mental health for individuals with these late-stage diseases.

Interestingly, late-stage glaucoma was not associated with VRQoL impediments. We hypothesize that this could possibly be because of differences in disease-specific treatment approaches. For instance, these patients could have had more aggressive IOP-lowering therapy, e.g., trabeculectomy, thereby reducing their reliance on IOP lowering eye drops that have been linked with substantial decrements in QoL due to visual and ocular discomfort^51,52^, hence resulting in paradoxical improvements in QoL in advanced stages of the disease. Moreover, IVI is a generic VRQoL questionnaire that measure the holistic impact of vision loss on QoL. As individuals with late-stage glaucoma usually still have good central vision, the use of such generic vision-specific questionnaires may not reflect the actual impediments experienced by these patients. Likewise, MMD is a complication of high/pathologic myopia that usually occurs gradually over time. As most of these patients are on long-term clinical follow-up, these individuals may be aware of or are informed regarding the potential likelihood of worsening of these degenerative changes over time^53^ and may hence be more emotionally prepared, which translates to better emotional well-being scores in late-stage disease. Last, our findings of no associations between late-stage glaucoma and MMD with VRQoL, could likely be due to the low number of late glaucoma (n = 26) and MMD (n = 7) cases, which is not sufficient to detect the observed effect sizes according to our sample size calculations (refer to the Supplementary Table S2 for the sample size requirement for each eye disease) and limits our ability to properly assess these associations.

Although, several studies have shown the impact of early and late-stage eye diseases on VRQoL, most studies to-date have focused on the association of a single eye pathology (e.g., glaucoma) and VRQoL, oftentimes confounded by the presence of co-morbid eye pathologies^21–23,54–56^. Our study offers, for the first time, a glimpse into the impact of these four age-related eye diseases on VRQoL, untainted by such confounders. By comparing the associations across these eye diseases, we have confirmed the differential impact of these eye diseases across three different domains of VRQoL. However, the IVI and NEI-VFQ-25/-50^57,58^, being generic VRQoL questionnaires, may not optimally reflect the specific person-centred impact of the different eye diseases and their associated interventions (e.g., difficulties arising from eye-specific symptoms and treatment-related effects). The difficulty in assessing such patient-reported difficulties with currently available VRQoL questionnaires advocate for the development and validation of disease-specific PROMs. Such PROMs, developed using modern psychometric methods including item banking and computerized adaptive testing (CAT), can quickly and accurately assess the impact of specific eye diseases on QoL across the disease spectrum. Our group has recently developed and validated DR-specific (RetCAT)^59,60^ and glaucoma (GlauCAT) item banks and CATs^61,62^, and is in the process of developing other disease-specific CATs including AMD (MacCAT) and myopia (MyoCAT), which may be promising tools for clinicians and eye clinics to carry out value-based evaluations of patient care.

Strengths of our study include the large population-based design in three large ethnic groups in Asia, the objective categorization of eye diseases using standardized disease grading protocols, ability to control for a range of risk factors including PVA, and the use of Rasch analysis to psychometrically validate and transform ordinal participant responses into interval-level measures to optimize measurement precision^63^. Limitations include the small number of participants with late stage eye disease, especially late AMD and late MMD, which might have reduced the power needed to detect statistically significant associations using our late stage disease categorization. Moreover, the small numbers precluded our ability to conduct ethnic-stratified analyses in our sample, so we are unable to tell if our observed associations differed across the Chinese, Malay and Indian ethnic groups^64^. Further studies to evaluate the ethnic-specific impact of early and late stage eye diseases on VRQoL are warranted. While it is possible that language/cultural differences in interpreting the IVI items may have affected the results, this impact is likely to be minimal, as the IVI questionnaire was administered to our multi-ethnic study participants by trained multi-lingual interviewers in English, Malay, Tamil, or Mandarin based on professional forward/backward translations of the instrument which was further checked by a panel of local speakers for cultural or linguistic issues. However, while the Chinese IVI has been formally validated^41^, the Tamil and Malay versions have not undergone a formal validation study and publication. As such, we are unable to provide validity and reliability indices for these two languages. Furthermore, as per our study protocol, not all participants undertook VF testing. As such, only a proportion of subjects with glaucoma had reliable VF data available, so we are unfortunately unable to look into combined visual efficiency (VA and VF) compared with VRQoL. We also did not collect data to study other aspects of visual function system such as contrast sensitivity, colour vision, depth perception, which could have been associated with our outcome. While it is possible that our findings would be affected if different thresholds to define early and late disease had been chosen, we believe that our objective assessment and standardized categorization of eye diseases will allow our study findings to be compared with that of other studies. It would be interesting to explore the associations of disease sub-types, such as polypoidal choroidal vasculopathy in the AMD group and pseudo-exfoliation glaucoma in the glaucoma group, with VRQoL. Future research using disease sub-types are needed to provide a more robust assessment of the impact of eye diseases on VRQoL. Although IVI is commonly used to measure the impact of VI resulting from any eye condition on QoL, it may not optimally detect QoL issues relating to specific eye conditions, e.g., glaucoma, which may explain the non-significant impact of glaucoma on VRQoL^65^. Last, while it would have been clinically interesting to assess the different impact of single vs. multiple eye diseases on VRQoL, the small number of participants with multiple eye diseases meant that this comparison was not possible. Future studies with adequately powered sample sizes are needed to evaluate the different impact of single vs. multiple eye diseases on VRQoL in order to further expand the knowledge base on the VRQoL impact of these eye diseases, and inform management strategies for individuals with multiple ocular comorbidities.

In conclusion, in our multi-ethnic Asian population we demonstrated a differential impact of early and late stages irreversible eye diseases on VRQoL domains. For early disease stages, there was no difference in the VRQoL outcomes between the four diseases. For late stages, VTDR followed by AMD appear to be the diseases with the greatest impact on VRQoL. In contrast, late-stage glaucoma and MMD did not affect VRQoL as captured by the IVI scale. Our study advocates the need for rehabilitative interventions to improve mobility, reading and mental health for individuals with late-stage diseases and the use of disease-specific PROMs to accurately measure the impact of specific eye diseases on QoL across the disease spectrum leading to personalized treatment strategies for management of different eye diseases.

## Supplementary Information

Supplementary Information.
