# Supplementary material for: Different impact of early and late stages irreversible eye diseases on vision-specific quality of life domains
Source: Sci Rep. 2022 May 19;12:8465. doi: 10.1038/s41598-022-12425-9 (PMC9120442; doi:10.1038/s41598-022-12425-9)
Supplement: Supplementary file 1 — Supplementary Information. [file 41598_2022_12425_MOESM1_ESM.docx]

**Different Impact of Early and Late Stages Irreversible Eye Diseases on Vision-specific Quality of Life Domains**

1. **Appendix 1.** Supplementary Methods
2. **Appendix 2.** Supplementary Results
3. **Appendix 3.** Supplementary Tables

**Appendix 1.** Supplementary Methods

*Assessment of the psychometric properties of the IVI using Rasch analysis*

Rasch analysis was undertaken to assess the psychometric properties of the IVI using the Andrich rating scale model^1^ with Winsteps software (version 4.6.0, Chicago, Illinois, USA). Rasch analysis is a form of Item Response Theory where ordinal questionnaire scores are transformed to estimates of interval measures (expressed in log of the odds units, or logits).^2^ Rasch analysis also provides a thorough exploration of the psychometric properties of the scale and a means to optimise them if needed, including response category functioning, measurement precision, item ‘fit’ to the underlying construct (e.g. emotional well-being), unidimensionality (i.e. measurement of a single construct), targeting of item difficulty to subjects’ level of impairment, and differential item functioning (DIF)/item bias.^3^

As the IVI has previously been shown to be multidimensional in the Singapore Chinese Eye Study (SCES),^4^ we conducted Rasch analysis separately on the three IVI scales (**Supplementary** **Table 1**). The Reading and Accessing Information scale initially demonstrated reasonable fit to the Rasch model, with good precision (range-based Person Reliability [PR] 0.97), ordered thresholds, unidimensionality, and no DIF for age or gender. However, item 1 and item 2 displayed misfit (infit MnSq>1.3) and were deleted. Subsequently, Item 14 displayed minor misfit (infit MnSq=1.39) but was retained due to perceived importance of the item content.

The Mobility and Independence scale displayed good precision (range-based PR 0.96), ordered thresholds, unidimensionality, and no DIF for age or gender. However, item 4 and item 5 displayed misfit (infit MnSq>1.3) and were deleted, which improved fit statistics overall.

The Emotional well-being scale had good precision (range-based PR 0.96), ordered thresholds, unidimensionality, and no DIF for age or gender. While item 29 displayed misfit (infit MnSq 1.41), it was retained due to perceived importance of item content.

Targeting of all three scales was suboptimal, with the participants, on average, too able for the items in the scale (difference between person and item means >4 logits). However, this is understandable given than most of the sample did not have vision impairment.

**Appendix 2.** Supplementary Results

**Power calculation to detect effect estimates for each eye disease**

**Supplementary Tables S2-S4** shows the power to detect effect estimates for each eye condition with the current sample size, as well as the sample size needed for each eye condition using study effect estimates with 80% power and 5% significance level. We found that aside from VTDR, the number of individuals with each late stage eye disease is below the number needed to achieve sufficient power to detect significance in the observed QoL reductions. This is particularly true for late AMD, where although the estimated effect on Emotional well-being is of a clinically important magnitude, three times as many cases are needed to achieve statistical power of at least 80%.

Considering the small number of cases of individuals with late stage eye diseases, statistical inferences drawn based on asymptotic assumptions of normality were checked. First, we verified visually that residuals of the multiple linear regression models did not show marked deviations from normality. Second, we performed a permutation test **(Supplementary Tables S2-S4),** which does not require any parametric assumptions. Comparison of the results of both the t-tests and permutation tests were very similar, suggesting that the conclusions drawn about statistical significance are robust.

**Unadjusted association between eye disease severity and VRQoL**

**Early stage**

In the unadjusted analysis for early-stage eye disease, compared to those with no eye disease, we found significantly lower Emotional well-being for people with early MMD (β -0.47; 95%CI -0.71 to -0.23), and decrements in Mobility for people with non-VTDR (β -0.25; 95%CI -0.45 to -0.05) and early AMD (β -0.17; 95%CI -0.32 to -0.02). Unadjusted pairwise comparisons of VRQoL outcomes between early stages of the four eye diseases showed no significant differences (all P>0.05; **Supplementary** **Table S5**).

**Late stage**

In the unadjusted analysis for late-stage disease, compared to controls, people with VTDR had decrements in Emotional well-being (β -1.10; 95%CI -1.58 to -0.62), Mobilty (β -0.67; 95%CI -1.03 to -0.30) and Reading scores (β -0.72; 95%CI -1.13 to -0.31), while late AMD was associated with the highest decrement in Mobility (β -0.95; 95%CI -1.63 to -0.28) compared to controls. Unadjusted pairwise comparisons of VRQoL outcomes between late stages of the four eye diseases showed that individuals with VTDR had significantly larger decrements in Emotional well-being (β -0.85; 95%CI -1.51to -0.19) and Reading (β -0.58; 95%CI -1.14 to -0.02) compared to those with late-stage glaucoma (**Supplementary Table S6**).

**Multivariable-adjusted association** (adjusting for fewer variables including age, gender, ethnicity, low SES, BMI, current smoking and presence of systemic diseases) **between eye disease severity and VRQoL**

**Early stage**

In the multivariable analysis adjusting for fewer variables (age, gender, race, low SES, BMI, current smoking, any systemic disease and PVI in the better eye) for early-stage eye disease compared to those with no eye disease, we found significantly lower Emotional well-being for people with early AMD (β -0.23; 95%CI -0.43 to -0.03), early glaucoma (β -0.46; 95%CI -0.88 to -0.04) and early MMD (β -0.36; 95%CI -0.61 to -0.12), and decrements in Mobility in people with non-VTDR (β -0.20; 95%CI -0.40 to -0.00). Reading was not associated with any early-stage eye disease. Multivariable adjusted pairwise comparisons of VRQoL outcomes between early stages of the four eye diseases showed that individuals with non-VTDR had significantly larger decrements in Mobility (β -0.28; 95%CI -0.55to -0.01) compared to those with early MMD (**Supplementary Table S7**).

**Late stage**

In the multivariable analysis adjusting for fewer variables (age, gender, race, low SES, BMI, current smoking, any systemic disease and PVI in the better eye), for late-stage disease compared to controls, people with VTDR had the highest decrement in Emotional well-being (β -1.13; 95%CI -1.60 to -0.65) and Reading scores (β -0.65; 95%CI -1.06 to -0.25). Late AMD was associated with the highest decrement in Mobility compared to controls, followed by VTDR (β -0.88; 95%CI -1.55 to -0.22 vs β -0.61; 95%CI -0.98 to -0.25). In contrast, late-stage glaucoma and MMD were not associated with any VRQoL decrements captured by the IVI scale. Multivariable adjusted pairwise comparisons of VRQoL outcomes between late stages of the four eye diseases showed that individuals with VTDR had significantly larger decrements in Emotional well-being compared to those with late-stage glaucoma (β -0.81; 95%CI -1.47 to -0.16) and late MMD (β -1.16; 95%CI -2.15 to -0.17), and greater Reading decrements compared to late-stage glaucoma (β -0.66; 95%CI -1.22 to -0.10). Individuals with late AMD had significantly larger IVI Mobility decrements than late glaucoma (β -0.77; 95%CI -1.52 to -0.05; **Supplementary Table S8**).

**Appendix 3.** Supplementary Tables

| Supplementary Table S1. Psychometric properties of the IVI Reading and Accessing Information; Mobility and Independence; and Emotional well-being scales | | | | | | |
| --- | --- | --- | --- | --- | --- | --- |
| Parameters | Rasch model | Reading | Reading (revised) | Mobility | Mobility (Revised) | Emotional |
| Items |  | 1-3, 6-7, 9-15 | 3, 6-7, 9-15 | 4-5, 8, 16-24 | 8, 16-24 | 25-32 |
| Disordered thresholds | No | No | No | No | No | No |
| Person reliability (range-based)* | >0.8 | 0.97 | 0.97 | 0.96 | 0.96 | 0.96 |
| PCA, variance by 1^st^ factor | >50% | 55.8% | 58.2% | 58.9% | 60.1% | 57.5% |
| PCA, Eigenvalue for 1^st^ contrast | <2.0 | 1.81 | 1.70 | 1.95 | 1.85 | 2.1 |
| Item fit (infit MnSq) | 0.7-1.3 | **Item 1 (1.74)**  **Item 2 (1.41)** | **Item 14 (1.39)** | **Item 5 (1.56); Item 4 (1.50)** | None | **Item 29 (1.41)** |
| Differential Item Functioning (DIF)§ | <1.0 logits and p>0.05 | None | None | None | None | None |
| Targeting, difference between person & item means | <1.0 logits | **4.69** | **5.01** | **4.95** | **5.18** | **5.33** |
| §DIF was assessed for age group (<60, ≥60); and gender  *As many participants were unaffected by VI in this population-based study, we calculated a range-based person-reliability coefficient using the person measure standard errors generated during Rasch analysis to manually compute how many statistically different levels of performance can be identified.^4^  PCA=Principal Components Analysis; Bolded values indicate misfit to the Rasch model; Shaded columns indicate the optimal fit obtained after making modifications to the scale based on Rasch fit statistics | | | | | | |

| **Supplementary Table S2.** Power calculation to detect effect estimates for each eye disease for Emotional well-being | | | | | | | |
| --- | --- | --- | --- | --- | --- | --- | --- |
| **Emotional** | | | | | | | |
|  | **Mean ± SE** | **Beta* (95% CI)** | **P** | **P2^** | **%** | **Power†** | **SS needed‡** |
| **Early stage disease** |  |  |  |  |  |  |  |
| None | 5.50 ± 0.03 | Reference |  |  |  |  |  |
| Non-VTDR | 5.42 ± 0.13 | -0.08 (-0.34 to 0.18) | 0.551 | 0.530 | -1.46 |  |  |
| Early AMD | 5.27 ± 0.10 | -0.23 (-0.43 to -0.03) | **0.024** | **0.022** | -4.15 |  |  |
| Early glaucoma | 5.04 ± 0.21 | -0.46 (-0.88 to -0.04) | **0.031** | **0.032** | -8.36 |  |  |
| Early MMD | 5.14 ± 0.12 | -0.36 (-0.61 to -0.12) | **0.003** | **0.004** | -6.61 |  |  |
| **Late stage disease** |  |  |  |  |  |  |  |
| None | 5.50 ± 0.03 | Reference |  |  |  |  |  |
| VTDR | 4.37 ± 0.24 | -1.13 (-1.61 to -0.66) | **<0.001** | **0.002** | -20.63 | 99.4 | 8 |
| Late AMD | 4.81 ± 0.44 | -0.69 (-1.56 to 0.18) | 0.122 | 0.094 | -12.51 | 33.0 | 21 |
| Mild-severe glaucoma | 5.18 ± 0.23 | -0.32 (-0.78 to 0.14) | 0.169 | 0.182 | -5.83 | 27.2 | 97 |
| Late MMD | 5.53 ± 0.44 | 0.03 (-0.84 to 0.91) | 0.939 | 0.922 | 0.62 | 5.1 | - |
| Adjusted for age, gender, race, education, income, housing, BMI, smoking status, alcohol use, diabetes, presence of any systemic disease and presenting VI in the better eye  Systemic diseases include diabetes, hypertension, hyperlipidemia, CVD and CKD  ^ Permutation test with 500 replicates  † Power to detect effect size with current sample size  ‡ Sample size needed to detect observed effect size with 80% power and 5% significance level | | | | | | | |

| **Supplementary Table S3.** Power calculation to detect effect estimates for each eye disease for Mobility | | | | | | | |
| --- | --- | --- | --- | --- | --- | --- | --- |
| **Mobility** | | | | | | | |
|  | **Mean ± SE** | **Beta* (95% CI)** | **P** | **P2^** | **%** | **Power†** | **SS needed‡** |
| **Early stage disease** |  |  |  |  |  |  |  |
| None | 5.48 ± 0.02 | Reference |  |  |  |  |  |
| Non-VTDR | 5.29 ± 0.10 | -0.19 (-0.39 to 0.01) | 0.059 | 0.064 | -3.53 |  |  |
| Early AMD | 5.45 ± 0.07 | -0.04 (-0.19 to 0.11) | 0.627 | 0.588 | -0.68 |  |  |
| Early glaucoma | 5.30 ± 0.16 | -0.18 (-0.50 to 0.14) | 0.261 | 0.262 | -3.33 |  |  |
| Early MMD | 5.55 ± 0.09 | 0.07 (-0.12 to 0.25) | 0.464 | 0.482 | 1.26 |  |  |
| **Late stage disease** |  |  |  |  |  |  |  |
| None | 5.48 ± 0.02 | Reference |  |  |  |  |  |
| VTDR | 4.87 ± 0.18 | -0.61 (-0.97 to -0.25) | **0.001** | **0.002** | -11.14 | 91.0 | 18 |
| Late AMD | 4.61 ± 0.34 | -0.87 (-1.54 to -0.21) | **0.010** | **0.024** | -15.89 | 72.4 | 9 |
| Mild-severe glaucoma | 5.37 ± 0.18 | -0.12 (-0.46 to 0.23) | 0.512 | 0.560 | -2.12 | 10.4 | 551 |
| Late MMD | 5.16 ± 0.34 | -0.32 (-0.99 to 0.34) | 0.343 | 0.282 | -5.88 | 15.6 | 64 |
| Adjusted for age, gender, race, education, income, housing, BMI, smoking status, alcohol use, diabetes, presence of any systemic disease and presenting VI in the better eye  Systemic diseases include diabetes, hypertension, hyperlipidemia, CVD and CKD  ^ Permutation test with 500 replicates  † Power to detect effect size with current sample size  ‡ Sample size needed to detect observed effect size with 80% power and 5% significance level | | | | | | | |

| \| **Supplementary Table S4.** Power calculation to detect effect estimates for each eye disease for Reading \| \| \| \| \| \| \| \| \| --- \| --- \| --- \| --- \| --- \| --- \| --- \| --- \| \| **Reading** \| \| \| \| \| \| \| \| \|  \| **Mean ± SE** \| **Beta* (95% CI)** \| **P** \| **P2^** \| **%** \| **Power†** \| **SS needed‡** \| \| **Early stage disease** \|  \|  \|  \|  \|  \|  \|  \| \| None \| 5.29 ± 0.02 \| Reference \|  \|  \|  \|  \|  \| \| Non-VTDR \| 5.22 ± 0.11 \| -0.06 (-0.29 to 0.16) \| 0.579 \| 0.578 \| -1.20 \|  \|  \| \| Early AMD \| 5.26 ± 0.08 \| -0.03 (-0.20 to 0.14) \| 0.731 \| 0.726 \| -0.56 \|  \|  \| \| Early glaucoma \| 5.27 ± 0.18 \| -0.01 (-0.37 to 0.34) \| 0.935 \| 0.942 \| -0.28 \|  \|  \| \| Early MMD \| 5.25 ± 0.10 \| -0.04 (-0.24 to 0.17) \| 0.737 \| 0.738 \| -0.67 \|  \|  \| \| **Late stage disease** \|  \|  \|  \|  \|  \|  \|  \| \| None \| 5.29 ± 0.02 \| Reference \|  \|  \|  \|  \|  \| \| VTDR \| 4.63 ± 0.21 \| -0.66 (-1.07 to -0.26) \| **0.001** \| **0.006** \| -12.49 \| 89.5 \| 19 \| \| Late AMD \| 5.09 ± 0.38 \| -0.20 (-0.94 to 0.55) \| 0.604 \| 0.610 \| -3.71 \| 8.3 \| 216 \| \| Mild-severe glaucoma \| 5.29 ± 0.20 \| 0.00 (-0.38 to 0.39) \| 0.984 \| 0.918 \| 0.08 \| - \| - \| \| Late MMD \| 5.08 ± 0.38 \| -0.21 (-0.95 to 0.53) \| 0.577 \| 0.624 \| -4.00 \| 8.6 \| 194 \| \| Adjusted for age, gender, race, education, income, housing, BMI, smoking status, alcohol use, diabetes, presence of any systemic disease and presenting VI in the better eye  Systemic diseases include diabetes, hypertension, hyperlipidemia, CVD and CKD  ^ Permutation test with 500 replicates  † Power to detect effect size with current sample size  ‡ Sample size needed to detect observed effect size with 80% power and 5% significance level \| \| \| \| \| \| \| \| |
| --- | --- | --- | --- | --- | --- | --- | --- | --- | --- | --- | --- | --- | --- | --- | --- | --- | --- | --- | --- | --- | --- | --- | --- | --- | --- | --- | --- | --- | --- | --- | --- | --- | --- | --- | --- | --- | --- | --- | --- | --- | --- | --- | --- | --- | --- | --- | --- | --- | --- | --- | --- | --- | --- | --- | --- | --- | --- | --- | --- | --- | --- | --- | --- | --- | --- | --- | --- | --- | --- | --- | --- | --- | --- | --- | --- | --- | --- | --- | --- | --- | --- | --- | --- | --- | --- | --- | --- | --- | --- | --- | --- | --- | --- | --- | --- | --- | --- | --- | --- | --- | --- | --- | --- | --- | --- | --- | --- | --- | --- | --- | --- | --- | --- | --- | --- | --- | --- | --- | --- | --- | --- | --- | --- | --- | --- | --- | --- | --- |

| **Supplementary Table S5.** Unadjusted pairwise comparisons of each IVI domain between early stage eye diseases | | | | |
| --- | --- | --- | --- | --- |
|  | Beta coefficient (95% CI), P-value | | | |
| Reference group# | Early MMD | Non-VTDR | Early AMD | Early Glaucoma |
| **Emotional** | | | | |
| None | **-0.47 (-0.71 to -0.23) P < 0.001** | 0.06 (-0.20 to 0.32) P = 0.652 | -0.14 (-0.34 to 0.05) P = 0.157 | -0.38 (-0.80 to 0.04) P = 0.079 |
| Early MMD |  | 0.53 (0.19 to 0.88) P = 0.003 | 0.33 (0.03 to 0.63) P = 0.031 | 0.10 (-0.38 to 0.58) P = 0.695 |
| Non-VTDR |  |  | -0.20 (-0.52 to 0.12) P = 0.217 | -0.44 (-0.93 to 0.05) P = 0.081 |
| Early AMD |  |  |  | -0.24 (-0.70 to 0.22) P = 0.314 |
| **Mobility** | | | | |
| None | -0.11 (-0.30 to 0.07) P = 0.229 | **-0.25 (-0.45 to -0.05) P = 0.016** | **-0.17 (-0.32 to -0.02) P = 0.028** | -0.25 (-0.57 to 0.07) P = 0.131 |
| Early MMD |  | -0.13 (-0.40 to 0.13) P = 0.323 | -0.06 (-0.29 to 0.18) P = 0.638 | -0.14 (-0.50 to 0.23) P = 0.467 |
| Non-VTDR |  |  | 0.08 (-0.17 to 0.33) P = 0.526 | -0.00 (-0.38 to 0.38) P = 0.994 |
| Early AMD |  |  |  | -0.08 (-0.43 to 0.27) P = 0.652 |
| **Reading** | | | | |
| None | -0.02 (-0.23 to 0.18) P = 0.833 | -0.07 (-0.29 to 0.16) P = 0.547 | -0.02 (-0.19 to 0.15) P = 0.828 | -0.02 (-0.38 to 0.33) P = 0.898 |
| Early MMD |  | -0.05 (-0.34 to 0.25) P = 0.758 | 0.00 (-0.25 to 0.26) P = 0.979 | -0.00 (-0.40 to 0.40) P = 0.996 |
| Non-VTDR |  |  | 0.05 (-0.22 to 0.32) P = 0.716 | 0.05 (-0.37 to 0.46) P = 0.829 |
| Early AMD |  |  |  | -0.00 (-0.39 to 0.38) P = 0.982 |

| **Supplementary Table S6.** Unadjusted pairwise comparisons of each IVI domain between late stage eye diseases | | | | |
| --- | --- | --- | --- | --- |
|  | Beta coefficient (95% CI), P-value | | | |
| Reference group# | Early MMD | Non-VTDR | Early AMD | Early Glaucoma |
| **Emotional** | | | | |
| None | -0.12 (-1.01 to 0.76) P = 0.783 | -0.25 (-0.71 to 0.21) P = 0.295 | -0.58 (-1.46 to 0.31) P = 0.200 | **-1.10 (-1.58 to -0.62) P < 0.001** |
| Early MMD |  | -0.12 (-1.11 to 0.87) P = 0.810 | -0.45 (-1.70 to 0.79) P = 0.476 | -0.97 (-1.97 to 0.03) P = 0.057 |
| Non-VTDR |  |  | -0.33 (-1.32 to 0.66) P = 0.513 | **-0.85 (-1.51 to -0.19) P = 0.011** |
| Early AMD |  |  |  | -0.52 (-1.52 to 0.48) P = 0.308 |
| **Mobility** | | | | |
| None | -0.58 (-1.25 to 0.10) P = 0.094 | -0.31 (-0.66 to 0.05) P = 0.088 | **-0.95 (-1.63 to -0.28) P = 0.006** | **-0.67 (-1.03 to -0.30) P < 0.001** |
| Early MMD |  | 0.27 (-0.49 to 1.03) P = 0.485 | -0.38 (-1.33 to 0.58) P = 0.440 | -0.09 (-0.86 to 0.68) P = 0.820 |
| Non-VTDR |  |  | -0.65 (-1.41 to 0.11) P = 0.096 | -0.36 (-0.86 to 0.15) P = 0.163 |
| Early AMD |  |  |  | 0.29 (-0.48 to 1.05) P = 0.464 |
| **Reading** | | | | |
| None | -0.52 (-1.27 to 0.23) P = 0.175 | -0.15 (-0.54 to 0.25) P = 0.468 | -0.23 (-0.98 to 0.52) P = 0.550 | **-0.72 (-1.13 to -0.31) P = 0.001** |
| Early MMD |  | 0.38 (-0.47 to 1.22) P = 0.384 | 0.29 (-0.77 to 1.35) P = 0.590 | -0.20 (-1.06 to 0.65) P = 0.642 |
| Non-VTDR |  |  | -0.08 (-0.93 to 0.76) P = 0.846 | **-0.58 (-1.14 to -0.02) P = 0.044** |
| Early AMD |  |  |  | -0.49 (-1.35 to 0.36) P = 0.257 |

| **Supplementary Table S7.** Multivariable-adjusted (for fewer variables) pairwise comparisons of each IVI domain between early stage eye diseases | | | | |
| --- | --- | --- | --- | --- |
|  | Beta coefficient (95% CI), P-value | | | |
| Reference group# | Early MMD | Non-VTDR | Early AMD | Early Glaucoma |
| **Emotional** | | | | |
| None | **-0.36 (-0.61 to -0.12) P = 0.003** | -0.08 (-0.34 to 0.18) P = 0.551 | **-0.23 (-0.43 to -0.03) P = 0.024** | **-0.46 (-0.88 to -0.04) P = 0.031** |
| Early MMD |  | 0.28 (-0.07 to 0.63) P = 0.114 | 0.14 (-0.17 to 0.44) P = 0.379 | -0.10 (-0.57 to 0.38) P = 0.693 |
| Non-VTDR |  |  | -0.15 (-0.47 to 0.17) P = 0.361 | -0.38 (-0.87 to 0.11) P = 0.127 |
| Early AMD |  |  |  | -0.23 (-0.69 to 0.22) P = 0.319 |
| **Mobility** | | | | |
| None | 0.08 (-0.10 to 0.26) P = 0.395 | **-0.20 (-0.40 to -0.00) P = 0.049** | -0.04 (-0.19 to 0.11) P = 0.620 | -0.19 (-0.51 to 0.13) P = 0.238 |
| Early MMD |  | **-0.28 (-0.55 to -0.01) P = 0.039** | -0.12 (-0.35 to 0.11) P = 0.311 | -0.27 (-0.63 to 0.09) P = 0.142 |
| Non-VTDR |  |  | 0.16 (-0.08 to 0.41) P = 0.188 | 0.01 (-0.36 to 0.38) P = 0.957 |
| Early AMD |  |  |  | -0.15 (-0.50 to 0.19) P = 0.386 |
| **Reading** | | | | |
| None | -0.02 (-0.23 to 0.18) P = 0.833 | -0.07 (-0.29 to 0.16) P = 0.547 | -0.02 (-0.19 to 0.15) P = 0.828 | -0.02 (-0.38 to 0.33) P = 0.898 |
| Early MMD |  | -0.05 (-0.34 to 0.25) P = 0.758 | 0.00 (-0.25 to 0.26) P = 0.979 | -0.00 (-0.40 to 0.40) P = 0.996 |
| Non-VTDR |  |  | 0.05 (-0.22 to 0.32) P = 0.716 | 0.05 (-0.37 to 0.46) P = 0.829 |
| Early AMD |  |  |  | -0.00 (-0.39 to 0.38) P = 0.982 |
| Adjusted for age, gender, race, low SES, BMI, smoking, any systemic disease & PVI better eye | | | | |
| Systemic diseases include diabetes mellitus, hypertension, hyperlipidaemia, CVD & CKD | | | | |
| BMI=body mass index; CVD=cardiovascular disease; CKD=chronic kidney disease; PVI=presenting visual impairment | | | | |
| VTDR=vision threatening diabetic retinopathy; AMD=age related macular degeneration; MMD=myopic macular degeneration | | | | |

| **Supplementary Table S8.** Multivariable-adjusted (for fewer variables) pairwise comparisons of each IVI domain between late stage eye diseases | | | | |
| --- | --- | --- | --- | --- |
|  | Beta coefficient (95% CI), P-value | | | |
| Reference group# | Early MMD | Non-VTDR | Early AMD | Early Glaucoma |
| **Emotional** | | | | |
| None | 0.04 (-0.84 to 0.91) P = 0.934 | -0.31 (-0.77 to 0.14) P = 0.178 | -0.68 (-1.55 to 0.19) P = 0.127 | **-1.13 (-1.60 to -0.65) P < 0.001** |
| Early MMD |  | -0.35 (-1.33 to 0.63) P = 0.483 | -0.72 (-1.95 to 0.51) P = 0.254 | **-1.16 (-2.15 to -0.17) P = 0.021** |
| Non-VTDR |  |  | -0.36 (-1.35 to 0.62) P = 0.466 | **-0.81 (-1.47 to -0.16) P = 0.015** |
| Early AMD |  |  |  | -0.45 (-1.44 to 0.54) P = 0.375 |
| **Mobility** | | | | |
| None | -0.31 (-0.97 to 0.36) P = 0.369 | -0.11 (-0.46 to 0.24) P = 0.541 | **-0.88 (-1.55 to -0.22) P = 0.009** | **-0.61 (-0.98 to -0.25) P = 0.001** |
| Early MMD |  | 0.20 (-0.55 to 0.94) P = 0.605 | -0.58 (-1.52 to 0.36) P = 0.226 | -0.31 (-1.06 to 0.45) P = 0.423 |
| Non-VTDR |  |  | **-0.77 (-1.52 to -0.03) P = 0.042** | -0.50 (-1.00 to -0.00) P = 0.048 |
| Early AMD |  |  |  | 0.27 (-0.48 to 1.02) P = 0.481 |
| **Reading** | | | | |
| None | -0.21 (-0.95 to 0.53) P = 0.584 | 0.01 (-0.38 to 0.39) P = 0.971 | -0.21 (-0.95 to 0.53) P = 0.576 | **-0.65 (-1.06 to -0.25) P = 0.002** |
| Early MMD |  | 0.21 (-0.62 to 1.05) P = 0.614 | -0.00 (-1.05 to 1.04) P = 0.994 | -0.44 (-1.29 to 0.40) P = 0.300 |
| Non-VTDR |  |  | -0.22 (-1.05 to 0.61) P = 0.607 | **-0.66 (-1.22 to -0.10) P = 0.020** |
| Early AMD |  |  |  | -0.44 (-1.28 to 0.40) P = 0.303 |
| Adjusted for age, gender, race, low SES, BMI, smoking, any systemic disease & PVI better eye | | | | |
| Systemic diseases include diabetes mellitus, hypertension, hyperlipidaemia, CVD & CKD | | | | |
| BMI=body mass index; CVD=cardiovascular disease; CKD=chronic kidney disease; PVI=presenting visual impairment | | | | |
| VTDR=vision threatening diabetic retinopathy; AMD=age related macular degeneration; MMD=myopic macular degeneration | | | | |

**References**

1 Linacre J. M. *A user’s guide to Winsteps/Ministeps Rasch-Model Computer Programs. Program Manual 4.0.0.* Chicago, IL: MESA Press (2017).

2 Mallinson, T. Why measurement matters for measuring patient vision outcomes. *Optom Vis Sci* **84**, 675-682 (2007).

3 Lamoureux, E. & Pesudovs, K. Vision-specific quality-of-life research: a need to improve the quality. *American journal of ophthalmology* **151**, 195-197 e192, doi:S0002-9394(10)00733-6 [pii]

10.1016/j.ajo.2010.09.020 [doi] (2011).

4 Fenwick, E. K. *et al.* Assessment of the psychometric properties of the Chinese Impact of Vision Impairment questionnaire in a population-based study: findings from the Singapore Chinese Eye Study. *Quality of life research : an international journal of quality of life aspects of treatment, care and rehabilitation* **25**, 871-880, doi:10.1007/s11136-015-1141-1 (2016).
